# Impact of Training on General Practitioner’s Knowledge, Attitude and Practices Regarding Emergency Contraception in Hyderabad

**DOI:** 10.12669/pjms.295.4068

**Published:** 2013

**Authors:** Seema Bibi, Razia Mustafa Abbasi, Shazia Awan, Roshan Ara Qazi, Sanober Ashfaque

**Affiliations:** 1 Dr. Seema Bibi, FCPS, Department of Obstetrics & Gynaecology, Liaquat University of Medical & Health Sciences, Jamshoro, Sindh, Pakistan.; 2 Dr. Razia Mustafa Abbasi, FCPS, Department of Obstetrics & Gynaecology, Liaquat University of Medical & Health Sciences, Jamshoro, Sindh, Pakistan.; 3 Dr. Shazia Awan, DGO, Department of Obstetrics & Gynaecology, Liaquat University of Medical & Health Sciences, Jamshoro, Sindh, Pakistan.; 4 Dr. Roshan AraQazi, FCPS, Department of Obstetrics & Gynaecology, Liaquat University of Medical & Health Sciences, Jamshoro, Sindh, Pakistan.; 5 Dr. Sanober Ashfaque, MS, Department of Obstetrics & Gynaecology, Liaquat University of Medical & Health Sciences, Jamshoro, Sindh, Pakistan.

**Keywords:** Emergency Contraception, Family Planning Training, Knowledge Attitude and Practices

## Abstract

***Objectives:*** To elaborate the impact of family planning training on general practitioners’ knowledge, attitude and practices regarding emergency contraception.

***Methods:*** A cross sectional survey involving 270 general practitioners was conducted in Hyderabad from 1^st^ Oct to 31^st^ Dec 2010. Participants were divided into two groups on the basis of attending family planning training course after graduation and were interviewed face to face. Data was noted on questionnaire asking their knowledge, attitude and practices regarding emergency contraception. Data was analyzed on SPSS version 11. Student t-test was applied to compare the proportions among two groups.

***Results:*** Out of 270 general practitioners, male & female participants were 132 (48.9%) and 138 (51.1%) respectively. Mean experience as private general practitioner was 7.48 + 7.6 years. One third of the participants 84 (31.1%) have attended five days training course on family planning in the past, while 186 (69.9%) did not have any training. Source of training was government institutes 46(17%) and non government organization in 38 (14.1%) cases. Significant positive difference was noted on emergency contraception knowledge, attitude and use in group who attended family planning training.

***Conclusion:*** Educational intervention has a positive impact on health care provider’s knowledge, attitude and practices of emergency contraception.

## INTRODUCTION

Pakistan’s population has reached the mark of 180 millions, making it as the sixth most populous country with projected population of 314 million in the year 2050.^1^ The issues of low contraceptive prevalence, high and sustained unmet need of family planning is responsible for enormous number of unplanned pregnancies and induced abortions in Pakistan. It has been estimated that annual number of unplanned pregnancies is 03 millions and about 890,000 couples choose induced abortion, mostly by unskilled health care providers to get rid of those unwanted pregnancies which have serious socio-economic, physical & psychological health implications.^2^

Post coital emergency contraception (PCEC) is considered world wide as safe, cost effective and evidence based strategy to avoid unplanned pregnancies and their consequences. It is defined as the use of a drug or a device to prevent pregnancy after unprotected intercourse. In United States, use of EC resulted in 43% of total decline in the abortion rate between 1994 to 2004.^3^ Different methods are offered with proven efficacy including two doses of combined estrogen and progestin, progestin alone or insertion of an IUCD. In 2002, results from a multicenter WHO trial reported good efficacy with single dose (1.5 mg) of levonorgestrel taken up to 120 hours of unprotected intercourse.^4^ PCEC remains underutilized in both developed and underdeveloped countries, despite it efficacy, safety and availability as over the counter drug. Situation in Pakistan is nearly same, in spite of the fact that PCEC is now integral part of family planning services provided by public and private sector and public awareness campaigns have also been launched on television and newspapers. World wide lack of awareness on the part of patients and physicians are found as main barrier to use of PCEC. In Pakistan, studies involving family physicians, obstetrician & gynecologist and resident trainees report over all positive attitude but low provisions of PCEC because of major knowledge inaccuracies, fear of side effects and liability. Researchers have recommended educational interventions for pre-service and in-service health care providers in order to promote PCEC.^5^^,^^6^

General practitioners are primary physicians providing health care at the doorstep of people and the first in line to whom women reach for their health related problems. Therefore this study was planned to find out the impact of short family planning training course on their knowledge, practice and attitude regarding emergency contraception. The data will help policy makers to plan their strategies for promoting family planning practices generally and post coital EC particularly, which is an important target in achieving MDG goals in Pakistan.

## METHODS

This cross sectional survey was conducted in district Hyderabad Sindh from 1^st^ Oct 2010 to 31^st^ Dec 2010. Two hundred seventy general practitioners (GPs) having more than one year experience of private general practice and who were willing to participate in survey were included. Consultants and residents of obstetrics & gynaecology were excluded. They were interviewed face to face by medical students and post graduate trainees after verbal informed consent. Participants were divided into two groups on the basis of attending /not attending short family planning training course after graduation. Knowledge, attitude and practices of participants among both groups were noted on two page questionnaire by asking different questions. Data was entered and analyzed on SPSS version 11. Student t-test was applied to compare the proportions (variable) between the two groups.

## RESULTS

A total of 270 general practitioners were interviewed. Frequency of male & female participants were approximately equal with 132 (48.9%) and 138 (51.1%) respectively. Mean experience as private general practitioner (GP) was 7.48 + 7.6 years. Nearly one third of the participants 84 (31.1%) have attended five days general family planning training course after medical graduation, while 186 (69.9%) did not received any training. Source of training was government institutes 46(17%) and non government organization (Green Star) in 38 (14.1%) cases. Table-I shows impact of training on general practitioners’ knowledge, attitude and practices regarding emergency contraception.

**Table-I:** Impact of training on GP’s knowledge, attitude and practices regarding emergency contraception

*Affirmative Response*	*Trained Group N=84*	*Un-Trained Group N=186*	*P-Value*
***Knowledge about***			
EC	83 (98.8%)	161 (86.6%)	< 0.001
Who need EC	83 (98.8%)	158 (84.9%)	< 0.0001
Diff. medical products	76 (90.5%)	126 (67.7%)	< 0.0001
Time interval	67 (79.8%)	67 (36.0%)	< 0.0001
Required dosage	71 (84.5%)	100 (53.8%)	< 0.0001
Mode of action	75 (89.3%)	113 (60.8%)	< 0.0001
***Attitudes***			
Feel comfortable if client request for EC	75 (89.3%)	124 (66.7%)	< 0.0001
Feel prescribing EC morally sound	67 (79.8%)	108 (58.1%)	< 0.001
***Practices***			
Prescribe EC	55 (65.5%)	76 (40.9%)	< 0.0001
Refer the client	44 (52.4%)	111 (59.7%)	< 0.28
Preferred medical products:			
Pills	55 (65.5%)	77 (44.4%)	< 0.0001
IUCD	10 (11.9%)	0	

## DISCUSSION

Results of this study revealed that over all knowledge of primary care physicians about concept, indication & proper dosage was not very poor, in line with national and international figures.^5^^-^^7^ However significant improvement in base line knowledge was observed in those general practitioners who have received training course on family planning. Educational intervention in the form of CME will be cost effective strategy for promoting family planning, particularly emergency contraception in our low resource setting.

Worldwide attitude of health care providers towards emergency contraception remains a big hurdle in its wide spread use. Shalini Singh and colleagues in their study conducted on 190 doctors in Delhi reported that half of the study population had the belief that easy availability of EC will promote promiscuity and will reduce the practice of regular contraceptive method use including condoms.^8^ Similarly a Pakistani study among family physicians found that promiscuity, religious constraints and liability were the main barriers for provision of EC^5^. Our study also supports above figures, though a positive impact on their attitude was observed following training, highlighting the importance of education in changing mindsets.

It is obvious from the findings of this study that imparting education to health care providers was likely to improve their knowledge, help in changing mindsets and increases the provision of family planning services. Similar trend was observed in an Indian Survey conducted on medical and paramedical staff of government dispensaries of south district in Delhi where training in contraception showed a positive & significant correlation with dispensing practice.^9^

Different training strategies have been tried to improve the knowledge of target population. It is found that interactive, close to practice training with adequate continuous support is more cost effective than traditional off site training courses.^10^^,^^11^ Charandabi and associates in a cluster randomized trial found peer education using existing human resources and infrastructure as a useful strategy for imparting education and improving performance in a large group of family planning service providers.^12^ At present there is no organized continuous medical education programme, offered by Pakistan medical licensing authority or medical universities for in-service health care providers of public & private sector, due to lack of policy and scarcity of human and financial resources. There is dire need that health policy managers should design & implement low cost diverse educational strategies targeting in-service as well pre-service health care providers, in order to improve the knowledge and thus quality of their medical services, including family planning.

***Strengths and limitations of the study: ***To the best of our knowledge this was the first community based survey assessing the impact of training on emergency contraception knowledge, attitude & practice of general practitioners of Sindh Province. However as the survey population is targeted the results could not be generalized. Second limitation of the study was use of convenient sampling which may lead to potential bias in the data. Properly designed randomized controlled trials including nurses, technicians & pharmaceutics may produce different & real picture.

## CONCLUSION

It is evident that educational interventional has a positive impact on health care provider’s knowledge, attitude and practice of emergency contraception. Low cost targeted strategies aimed to improve the health care providers’ technical knowledge as well as behavior change & communication skills are essential to improve the existing situation related to family planning services.
